# Pharmacodynamic Thresholds for Beta-Lactam Antibiotics: A Story of Mouse *Versus* Man

**DOI:** 10.3389/fphar.2022.833189

**Published:** 2022-03-18

**Authors:** Angela V. Berry, Joseph L. Kuti

**Affiliations:** Center for Anti-Infective Research and Development, Hartford Hospital, Hartford, CT, United States

**Keywords:** beta-lactam, pharmacodynamics, pre-clinical models, therapeutic drug monitoring, susceptibility breakpoints

## Abstract

Beta-lactams remain a critical member of our antibiotic armamentarium and are among the most commonly prescribed antibiotic classes in the inpatient setting. For these agents, the percentage of time that the free concentration remains above the minimum inhibitory concentration (%*f*T > MIC) of the pathogen has been shown to be the best predictor of antibacterial killing effects. However, debate remains about the quantity of *f*T > MIC exposure needed for successful clinical response. While pre-clinical animal based studies, such as the neutropenic thigh infection model, have been widely used to support dosing regimen selection for clinical development and susceptibility breakpoint evaluation, pharmacodynamic based studies in human patients are used validate exposures needed in the clinic and for guidance during therapeutic drug monitoring (TDM). For the majority of studied beta-lactams, pre-clinical animal studies routinely demonstrated the *f*T > MIC should exceed approximately 40–70% *f*T > MIC to achieve 1 log reductions in colony forming units. In contrast, clinical studies tend to suggest higher exposures may be needed, but tremendous variability exists study to study. Herein, we will review and critique pre-clinical versus human-based pharmacodynamic studies aimed at determining beta-lactam exposure thresholds, so as to determine which targets may be best suited for optimal dosage selection, TDM, and for susceptibility breakpoint determination. Based on our review of murine and clinical literature on beta-lactam pharmacodynamic thresholds, murine based targets specific to each antibiotic are most useful during dosage regimen development and susceptibility breakpoint assessment, while a range of exposures between 50 and 100% *f*T > MIC are reasonable to define the beta-lactam TDM therapeutic window for most infections.

## Introduction

Beta-lactams are among the most prescribed antibiotic classes in the hospital setting, as these drugs are frequently used as prophylaxis and as empiric therapy for pneumonia, bloodstream infections, intra-abdominal infections, and infections of the urinary tract (Holten and Onusko, 2000; Bush and Bradford, 2016). For instance, penicillin-based antibiotics are the most frequently used antibiotic class in the ICU and accounted for 36% of all antibiotic use (Vincent et al., 2020). In support of their robust utilization, beta-lactams are frequently recommended by national and international treatment guidelines, and have been prescribed for many indications that correspond with their utilization (Holten and Onusko, 2000; Guilhaumou et al., 2019).

Despite their robust indications and frequent utilization, therapeutic failures among beta-lactam antibiotics, like any antibiotic, are not completely unavoidable. Reasons for clinical failure are often related to host issues (e.g., source control, underlying co-morbidities, and severity of infection) or lack of optimal exposure due to lower than anticipated concentrations, bacterial resistance, or both. Moreover, the variability in beta-lactam pharmacokinetics that can be observed across certain patient populations suggests additional efforts are sometimes required to ensure optimal exposure, even against susceptible organisms. Therapeutic drug monitoring (TDM) is one approach used clinically to optimize drug exposure while minimizing toxicity (Reeves et al., 2016). Currently, beta-lactam TDM is relatively uncommon, but there is gaining interest in its role and implementation given the diversity of indications and patient types for which these drugs are utilized (Roberts et al., 2010). TDM is also performed for other antimicrobials, including aminoglycosides, voriconazole, and vancomycin (Abdul-Aziz et al., 2020).

Well before a beta-lactam is ever available for prescription to patients, however, pre-clinical models are conducted to understand *in vivo* efficacy and establish exposure response relationships (Waack et al., 2020). These preclinical models, including the classic neutropenic murine thigh infection model, demonstrate the killing profile of the antibiotic versus a pathogen (concentration-dependent versus concentration-independent (i.e., time-dependent) killing, the pharmacodynamic index that best predicts killing [e.g., time that free drug concentrations remain above the minimum inhibitory concentration (*f*T > MIC)], and quantify the exposure thresholds needed for specific microbiological endpoints. These data are paramount to successful establishment of an optimal dosing regimen for further clinical development, as well as support provisional susceptibility breakpoints (Bulitta et al., 2019). Dosing regimens developed based on supportive pre-clinical animal models increases the likelihood that drug candidates are successful in phase 3 clinical trials. Once approved and available for clinical use, beta-lactam antibiotics are then often prescribed to treat infections in patient populations not included in original phase 3 studies, as well as for organisms resistant to prior standard of care. As such, these more challenging indications for use of beta-lactams beg the question of whether approved dosing regimens remain sufficient and if similar pharmacodynamic thresholds apply. Therefore, human pharmacodynamic studies are requisite to confirm pre-clinical observations and to further guide management of serious infections in critically ill patients *via* TDM. Herein, we will summarize and critique pre-clinical and human-based pharmacodynamic studies of beta-lactams developed after year 2000 with the aim of comparing beta-lactam exposure thresholds required to achieve defined endpoints. We searched Pubmed/Medline with search terms (beta-lactams, pharmacodynamics, murine, thigh-infection, pneumonia, and time above the MIC). We identified all beta-lactams approved after 2000 and restricted studies to these agents for our murine review. We included all human studies for any beta-lactam published after year 2000. The citation list of included papers was also reviewed to identify studies missed during initial search.

## Beta-Lactam PK/PD: Lessons From Pre-Clinical Models

**FIGURE 1:**
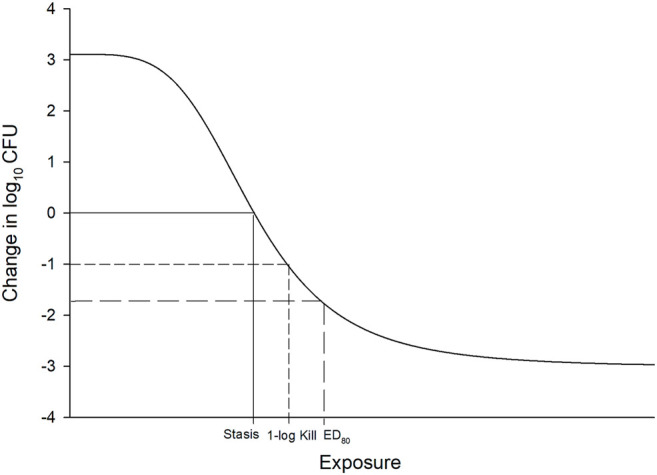
Example of sigmoidal Emax model used to define exposure response relationship in pre-clinical and clinical studies. The *x*-axis is typically drug exposure (e.g., dose, concentration, T > MIC) and the *y*-axis is effect (i.e., change in log10 CFU from baseline). Stasis, 1 log_10_ CFU reduction, and ED80 requirements are demonstrated.

*In vitro* and *in vivo* animal studies consistently demonstrate that *f*T > MIC is the pharmacodynamic parameter that best describes the killing profile of beta-lactam antibiotics (Vogelman and Craig, 1986; Reed, 2000; Lodise et al., 2006). In fact this has been established since the first beta-lactam was discovered. Eagle and others first demonstrated that longer “continuous” infusions of penicillin G led to improved microbiologic success against *Streptococcus pyogenes* in a mouse infection model in 1953 (Eagle et al., 1953). But it wasn’t until the 1980s that Craig and Ebert (Leggett et al., 1989) introduced the neutropenic murine thigh and lung infection models tools to identify thresholds required for specific antibacterial endpoints. Exposures generated from these experiments were analyzed using a non-linear concentration (or dose) response model, referred to as the maximal effect (Emax) and sigmoidal Emax model (Holford, 2017). Fitting a model to the exposure effect data leads to reasonable estimates of effect of increasing exposure (Figure 1).

The most common endpoints utilized in these pharmacodynamic studies include the exposure required for stasis, 1-log_10_ reductions in colony forming units (CFU), and 2-log_10_ CFU reductions after 24 h on the Emax curve. Alternative endpoints such as the effective dose (ED) or exposure (EE) that reduces the bacterial population by a certain percentage (e.g., 50, 80, and 90%) from baseline have also been proposed. However, based on guidance by the European Medicine’s Agency (EMA) and Food and Drug Administration (FDA), most antibiotic developers seek to develop a dosage that provides the most rapid and greatest amount of killing in the model, but this must be balanced with risks of toxicity and cost of goods (Bulitta et al., 2019). Notably, many antibiotics are incapable of achieving ≥2 log_10_ reductions over 24 h if they are “slower killing” agents or have higher MICs against a specific targeted pathogen. As a result, static and 1 log_10_ CFU reductions are the most referenced thresholds used during pre-clinical development, with stasis generally reserved for less serious infections such as complicated urinary tract infections and acute bacterial skin and skin structure infection, while ≥1-log_10_ CFU reductions are targeted for more serious infections including pneumonia and bloodstream infections. The application of these pharmacodynamic thresholds combined with Monte Carlo simulation to identify optimal dosing of antibiotics for human studies has led to successful application to the FDA for respiratory tract infections (Bulik et al., 2013).

The earliest pre-clinical studies of beta-lactam antibiotics partitioned these drugs by their ring structure class. Against Gram-negative and many Gram-positive bacteria, cephalosporin based beta-lactams typically required 60–70% *f*T > MIC for 1–2 log_10_ CFU reductions and 30–40% *f*T > MIC for stasis (e.g., cefotaxime against *Klebsiella pneumoniae* in the neutropenic murine lung infection model) (Craig, 1998). In contrast, penicillin based beta-lactams generally required less (i.e., 50–60% *f*T > MIC) for maximal effects, while carbapenems required approximately 40% *f*T > MIC due to a longer post antibiotic effect (Turnidge, 1998). Similar reductions in static exposure requirements were observed.

### Contemporary Beta-Lactams

Studies over the last 20 years have validated these observations in the neutropenic murine infection models. The efficacy and exposure-response of ceftaroline, the first approved cephalosporin with activity against methicillin-resistant *Staphylococcus aureus* (MRSA), was first evaluated in the murine thigh and lung infection models against *S. aureus*, *Streptococcus pneumoniae*, *Escherichia coli*, and *K. pneumoniae* (Andes and Craig, 2006). Stasis, 1-log_10_, and 2-log_10_ CFU reductions were observed at ceftaroline exposures of 26 ± 8%, 33 ± 9%, and 45 ± 13% *f*T > MIC against the *S. aureus* isolates, respectively. These values were 39 ± 9%, 43 ± 9%, and 50 ± 10% for *S. pneumoniae*. Finally against the Gram-negative organisms combined, 28 ± 9% *f*T > MIC was required for stasis, and 41 ± 11% was required for 1 log_10_ CFU reductions. Notably, 2 log_10_ reductions were not achieved for all Gram-negative isolates, although a mean of 54% was reported for the isolates that did. Later, ceftaroline was evaluated as a human simulated regimen (equivalent to 600 mg q12h) in the lung infection model. Seventeen *S. aureus* isolates (2 MSSA, 15 MRSA) with ceftaroline MICs between 0.5 and 4 mg/L were studied. The composite Emax curve identified 16% *f*T > MIC as the threshold required for stasis and 41% *f*T > MIC for $\mathrm{1-lo}g_{10}$ CFU reductions (Bhalodi et al., 2012), values similar to the original dose fractionation studies by Andes and others. A neutropenic thigh model using the same ceftaroline human simulated regimen against 26 *S. aureus* isolates (4 MSSA, 22 MRSA) with ceftaroline MICs between 0.125 and 4 mg/L identified static and 1 log_10_ CFU reduction thresholds of 19.3 and 27.5%, respectively (Keel et al., 2011).

Studies conducted of a second anti-MRSA cephalosporin, ceftobiprole, which is approved in Europe and Canada, but not yet the United States, demonstrated bacteriostatic effects when the *f*T > MIC was 36–45%, 14–28%, and 15–22% against Enterobacterales*, S. aureus*, and *S. pneumoniae*, respectively, in the neutropenic thigh infection model (Craig and Andes, 2008). Although thresholds for 1 log_10_ reductions were not provided, the mean *f*T > MIC required for 2 log_10_ CFU reductions were 64.5, 29.3, and 25.8%, respectively. For isolates that also grew in the pneumonia model, plasma exposure requirements were identical between infection sites. Notably, this was because of very high penetration of ceftobiprole into the epithelial lining fluid of mice, a trait that did not translate to humans, thereby leading to poor pulmonary exposures during phase 3 studies and inferior clinical outcomes (Rodvold et al., 2009).

The pharmacodynamics of the potent anti-*Pseudomonas* cephalosporin, ceftolozane, was determined using the neutropenic thing infection model (Craig and Andes, 2013). Against wild-type *E. coli* and *K. pneumoniae*, stasis and 1-log CFU reductions were achieved at 26.3 ± 2.1% and 31.6 ± 1.65 T > MIC, respectively. These exposure requirements were 25.5 ± 2.8% and 31.5 ± 2.8% for *Pseudomonas aeruginosa*. While total drug exposures were reported in the study, protein binding was less than 5% in the mice. Clinically, ceftolozane is only available combined with tazobactam to add stability against hydrolysis from extended spectrum beta-lactamases (ESBL). Tazobactam was added in 2:1, 4:1, and 8:1 ratios to the ceftolozane doses and all data were analyzed together against ESBL producing Enterobacterales. Stasis and 1 log_10_ CFU reductions were observed at 31.1 and 34.8% T > MIC, respectively. Notably, the reported 1 log thresholds for ceftolozane were numerically lower than required for previous anti-*Pseudomonas* cephalosporins, so a follow up study including 14 *P. aeruginosa* with ceftolozane MICs ranging from 2–16 mg/L was conducted (Lepak et al., 2014). Stasis and 1 log thresholds were increased to 31.2 ± 6.9% and 39.4 ± 7.5% T > MIC, values still lower than historical cephalosporins that require 60–70% *f*T > MIC for similar activity. Although 2 log thresholds were reported to be ∼42% T > MIC, this was only achieved (i.e., Emax) against five of the 14 isolates, and is therefore not representable of all isolates included in the study.

Cefiderocol is the first approved siderophore cephalosporin antibiotic. Its pharmacodynamics were studied in a number of murine thigh and lung infection models against various multi-drug resistant Gram-negative pathogens of interest (Katsube et al., 2019). Against a wild-type *P. aeruginosa* (cefiderocol MIC: 0.25 mg/L, cefepime MIC: 1 mg/L), stasis and 1 log_10_ CFU reductions were achieved at 47.5 and 57.6% *f*T > MIC in the murine thigh infection model (Nakamura et al., 2019). For comparison, cefepime required 61.7 and 87.7% *f*T > MIC for similar activity against the isolate. Pharmacodynamic analyses of cefiderocol against additional Enterobacterales and *P. aeruginosa*, including carbapenem resistant strains, resulted in stasis requirements of 62.5 ± 27.4% and 63 ± 15.5% *f*T > MIC, respectively; 1 log_10_ CFU reductions were observed with *f*T > MIC of 73.3 ± 23.3% and 72.2 ± 21.4%. T > MIC requirements were greater for carbapenem resistant isolates (85.2%) compared with carbapenem susceptible isolates (61.3%). In the lung infection model, 1 log_10_ CFU reductions were achieved with thresholds of 64.4 ± 22.5%, 70.3 ± 9.0%, 88.1 ± 3.4% and 53.9 ± 18.1% *f*T > MIC against Enterobacterales, *P. aeruginosa*, *Acinetobacter baumannii*, and *Stenotrophomonas maltophilia*, respectively. A similar cefiderocol *f*T > MIC target (∼81%) was documented in a separate murine thigh infection model using against eight *P. aeruginosa* (Ghazi et al., 2018).

Carbapenems have historically been reported to require lower T > MIC thresholds compared with other beta-lactams due to a prolonged post-antibiotic effect. Over the last 20 years, two carbapenems were developed and successfully approved. Ertapenem pharmacodynamics in the murine thigh infection model observed stasis requirements of 19% (range: 2–38) *f*T > MIC against *E. coli* and *K. pneumoniae*, including ESBL producing strains (Maglio et al., 2005). Although 1 log_10_ reductions were not reported, the ED_80_ exposure requirement was 33% (range: 13–65) *f*T > MIC. Notably, exposure requirements were similar between ESBL producing and non-ESBL producing isolates. Doripenem was studied in the neutropenic murine thigh infection model against 24 clinical *P. aeruginosa* isolates with MICs ranging from 0.125 to 16 mg/L (Kim et al., 2008). Using an Emax curve comprised of data for all 24 *P. aeruginosa* isolates included, stasis, 1 log_10_, and 2 log_10_ CFU reductions were observed at ∼20, 30, and 40% *f*T > MIC, respectively. Similar exposure requirements have been reported for carbapenems against *Acinetobacter* spp. The pharmacodynamics of meropenem, imipenem, and doripenem were studied against 14 *A. baumannii*; stasis, 1 log_10_, and 2 log_10_ CFU reductions were achieved at 24, 33, and 48% *f*T > MIC (Macvane et al., 2014a). A separate thigh infection model identified meropenem *f*T > MIC requirements of 16.7% (range: 7.3–24.2) for stasis and 26.8% (14.5–36.9%) for 1 log_10_ CFU reductions against six *A. baumannii* (Sabet et al., 2020). Two log reductions were achieved in five of the six isolates at 41.6% (range 26–53.2%) *f*T > MIC.

### Beta-Lactam/Beta-Lactamase Inhibitor Combination Antibiotics

Many beta-lactams approved in the last decade, as well as the majority of those in pre-clinical development, have aimed to thwart beta-lactamase mediated resistance by combining an older beta-lactam backbone with a novel beta-lactamase inhibitor drug. Ceftazidime/avibactam, meropenem/vaborbactam, and imipenem/cilastatin/relebactam were developed to target multidrug resistant gram-negatives that had developed resistance to their beta-lactam backbone components. With their development, the science of beta-lactamase inhibitor pharmacodynamics has made significant advances (Monogue and Nicolau, 2019).

During development of these agents, the common assumption is that the pharmacodynamics of the backbone beta-lactam remains consistent with the agent when administered alone. Instead, the focus is on what inhibitor exposures are required to potentiate the MIC of the backbone. Meropenem T > MIC requirements for stasis, 1 log_10_ and 2 log_10_ reductions were 30, 35, and 45% *f*T > MIC against Gram-negatives in the neutropenic thigh infection model; however, vaborbactam required a free area under the curve to MIC (*f*AUC/MIC) of 38 to enable meropenem to achieve 1 log_10_ kill at 35% *f*T > MIC against *K. pneumoniae* producing the KPC-carbapenemase (Food and Drug Administration, 2021). For ceftazidime/avibactam, the values used during development were 50% *f*T > MIC for ceftazidime and free avibactam concentrations that remained above a critical concentration threshold of 1 mg/L for at least 50% of the dosing interval (50% *f*T > C_T_ 1 mg/L) (Das et al., 2019; Das et al., 2020). Indeed, studies in the neutropenic thigh infection model of humanized ceftazidime/avibactam (2g/0.5 g every 8 h as 2 h infusion) in lung epithelial lining fluid (ELF) demonstrated ≥1 log_10_ CFU reductions against *P. aeruginosa* with MICs up to 32/4 mg/L, which corresponded with ceftazidime exposures ≥19% *f*T > MIC in ELF (Housman et al., 2014). A subsequent study in the murine thigh and lung infection model identified ceftazidime exposures of 0–29% *f*T > MIC required for stasis, noting that the ceftazidime MICs of the study isolates were 32–128 mg/L. However, avibactam exposures of 46.9% *f*T > C_T_ 1 mg/L, when combined with a fixed ceftazidime dosage that resulted in 50% *f*T > ceftazidime/avibactam MIC, achieved 1 log kill against *P. aeruginosa* (Berkhout et al., 2016). Finally, for imipenem/cilastatin/relebactam, 40% *f*T > MIC was assumed to be the imipenem threshold used for 1 log_10_ CFU reductions, while relebactam exposure targets were *f*AUC/MIC of 3.3, 4.3, and 7.0 for stasis, 1 log_10_, and 2 log_10_ reductions against *P. aeruginosa* in the murine thigh infection model (Patel et al., 2020).

### Summary of Pharmacodynamic Thresholds From Pre-Clinical Murine Studies

For the beta-lactams developed and approved since year 2000, neutropenic murine thigh and lung infection models have consistently identified *f*T > MIC as the pharmacodynamic index associated with CFU reduction at 24 h; furthermore, required exposures have been similar to studies conducted since the 1980s. For the two cephalosporins targeted at Gram-positive bacteria (ceftaroline and ceftobiprole), *f*T > MIC of 25–40% was required for 1 log_10_ CFU reductions. For Gram-negative targeted cephalosporins (ceftolozane and cefiderocol), *f*T > MIC requirements for 1 log_10_ reductions encompassed a wider range of 30–40% for ceftolozane, 61% for cefiderocol against wild-type gram-negatives, and 85% for cefiderocol against carbapenemase producing Gram-negatives. The carbapenems (ertapenem and doripenem) achieved approximately 1 log_10_ CFU reductions with exposures of 30–40% *f*T > MIC. Finally, when combined with novel beta-lactamase inhibitors targeting enzyme mediated resistance, the pharmacodynamic threshold requirements for ceftazidime/avibactam, meropenem/vaborbactam, and imipenem/cilastatin/relebactam were consistent with the beta-lactam backbones alone. However, the pharmacodynamic index of each beta-lactamase inhibitor will vary by drug as well as which beta-lactam backbone it is combined with. With few exceptions, a range of 40–70% *f*T > MIC was observed for beta-lactams to achieve 1 log_10_ CFU reductions in the mouse infection models. These data were very useful in defining optimal dosing regimens during drug development and for support of susceptibility breakpoints.

Although mean exposure requirements are similar to those previously reported, strain to strain variability should be better appreciated. Even in these contemporary pharmacodynamic studies, coefficient of variation (CVs) around threshold requirements were routinely 20–30%. It should be noted, however, that exposure requirements exceeding 100% *f*T > MIC in specific isolates were absent from the majority of these studies. While pharmacokinetic variability among mice in these studies can account for some of this variability, differences within individual isolates still contribute to varying pharmacodynamic thresholds, as demonstrated in *in vitro* chemostat experiments where drug exposure can be quantified in each individual model (Grupper et al., 2019). As a result, some have suggested that the variability in thresholds observed should be accounted for alongside pharmacokinetic variability and natural MIC variation when conducting Monte Carlo simulations to guide optimal dosing and susceptibility breakpoints (Soon et al., 2013).

## Beta-Lactam PK/PD: Lessons From Clinical Studies

While original dosing for most antibiotics is primarily guided by pre-clinical studies, the use of these drugs in patient populations who were excluded from original regulatory clinical trials may complicate confidence in the dosing regimen. These patients may have different pharmacokinetics due to underlying illness, co-morbidities, or life-saving treatment modalities (Roberts et al., 2014a). They may have different infection sources or be infected with difficult to treat/more resistant pathogens. Collectively, these challenges are responsible for endorsement of beta-lactam TDM and individualized dosing (Roberts et al., 2010; Fratoni et al., 2021). The question then remains, if one is to pursue TDM, what exposures should be targeted to increase the likelihood of success? The pre-clinical exposure thresholds that generated many of the dosing strategies to begin with and have a part in influencing susceptibility breakpoints are a reasonable start. But clinical studies are valuable to validate or further guide exposure requirements aimed at achieving successful clinical response.

### Point Prevalence Studies

Critically ill patients receiving “standard” beta-lactam doses appear to be less likely to achieve pharmacodynamic thresholds; however, many of these studies use high exposure targets such as 100% T > MIC or even 100% T > 4x MIC (i.e., a minimum concentration that is four fold higher than the MIC for the entire duration of the dosing interval). The DALI (Defining Antibiotic Levels in Intensive Care Patients) study was a prospective, multicenter, pharmacokinetic point-prevalence study that sought to define beta-lactam exposure variability in 248 critically-ill patients (Roberts et al., 2014b). As this was among the first studies to quantify exposure attainment in critically ill patients, several thresholds were defined *a priori*, including 50 and 100% *f*T > MIC, as well as more aggressive thresholds of 50 and 100% *f*T>4xMIC. The investigators observed that 16% of infected patients did not achieve at least 50% *f*T > MIC, and 39.6% of all patients had exposures less than 100% *f*T > MIC. Patients who did not achieve at least 50% *f*T > MIC exposures were 32% less likely to have a positive clinical outcome. Although the authors preferred utilization of the more aggressive thresholds, the receiver operator curves (ROC) suggested no differences in predicting clinical failures between 50 and 100% *f*T > MIC.

The EXPAT trial, a prospective, observational study that included 147 ICU patients receiving various beta-lactams, found that 63.3% of patients achieved 100% *f*T > MIC while only 36.7% attained 100% *f*T>4xMIC (Abdulla et al., 2020). Lower exposure targets were not assessed. Male gender, a larger glomerular filtration rate (i.e., >90 ml/min/1.73m2), and larger body mass increased the risk of non-target attainment. However, neither threshold was significantly associated with 30 days survival. A number of additional point-prevalence type studies have demonstrated low rates of achieving beta-lactam exposure targets but none of these studies have attempted to link clinical outcomes with exposure attainment (Carlier et al., 2013; Fournier et al., 2015; Ehmann et al., 2017; Cies et al., 2018). A common theme in these studies is the absence of identified pathogens and their antibiotic MICs. When unavailable, investigators reasonably assumed worse-case scenario by substituting the susceptibly breakpoint or epidemiological cut-off (ECOFF). However, this potentially leads to underestimation of exposure, which may explain why high exposures were not associated with clinical response (i.e., actual MICs were much lower than the breakpoint and sufficient drug exposure was already achieved).

### Clinical Pharmacodynamic Studies

A number of studies specifically aimed at evaluating beta-lactam exposure response relationships in patient populations have been conducted. Most of these studies use covariate based population pharmacokinetic models (e.g., clearance equal to a proportion of creatinine clearance and volume of distribution calculated by body weight) to estimate pharmacokinetic parameters that are then used to calculate T > MIC. While a reasonable estimate, this is a limitation in contrast to determining observed concentration and pharmacokinetic profiles in individuals. Many studies also routinely include various infection types, use limited MICs derived from automated susceptibility testing (AST) systems, and varying clinical endpoints. These limitations must be taken into consideration when evaluating identified pharmacodynamic thresholds.

Crandon et al. (2010) evaluated cefepime pharmacodynamics in 56 patients with pneumonia (66%), skin and skin structure infections (25%), and bacteremia (9%). Using a covariate based model to estimate pharmacokinetics, but actual isolate MICs determined by Etest, they identified exposures >60% *f*T > MIC to be significantly associated with a positive microbiological response. In 180 patients with Gram-negative bloodstream infections (most commonly *E. coli*, *P. aeruginosa*, and *K. pneumoniae*), cefepime exposures of 68–74% *f*T > MIC were associated with a higher odds of survival (Rhodes et al., 2015). The study is strengthened by its larger numbers, survival endpoint, and the use of two different covariate based models to estimate cefepime pharmacokinetics independently; however, MICs were derived from a VITEK AST system with limited range between 1 and 64 mg/L. T > MIC thresholds of 43 and 53% were identified in two additional studies evaluating cefepime and ceftazidime in patients with nosocomial pneumonia; both studies also utilized covariate based pharmacokinetic models to estimate exposures (Muller et al., 2013; Macvane et al., 2014b). Finally, piperacillin/tazobactam pharmacodynamics was assessed in a retrospective study of 78 patients with *P. aeruginosa* bacteremia (Tannous et al., 2020). The investigators employed a covariate based population pharmacokinetic model and identified *f*T > MIC of >60.9% to be associated with in-hospital survival.

A few studies with cephalosporins suggest higher exposure thresholds. Tam and others evaluated 20 patients treated with cefepime plus an aminoglycoside who were infected with various Gram-negatives from various sources (Tam et al., 2002). The investigators used observed cefepime concentrations to calculate exposure, and MICs were conducted *via* Etest. Microbiological success was 89% when T > MIC was at least 100%, and 0% when it was less than 100% (*p* = 0.032); however, the range of exposures was not described. When Classification and Regression Tree (CART) was employed, a minimum concentration (Cmin)/MIC ratio of 4.3 was significantly associated with microbiological success. The final logistic regression model predicted 80 and 90% success when T > 4.3 x MIC was 83 and 95% of the dosing interval. A follow up study in 33 patients with pneumonia found similar exposure requirements for cefepime (Aitken et al., 2015). In contrast to the original study, the latter used a covariate population model to estimate cefepime pharmacokinetics and MICs from VITEK. The majority of patients had *P. aeruginosa* (*n* = 17) followed by *Acinetobacter baumannii* complex (*n* = 5), but the highest observed MIC was 8 mg/L thus 32/33 patients were reported to have achieved 100% *f*T > MIC. As a result, clinical failure (12/33) was most significantly correlated with a *f*Cmin/MIC ratio <2.1. Gatti and others reported a case series of 13 patients treated with cefiderocol for extensively drug resistant *A. baumannii* bloodstream infections or ventilator associated pneumonia (Gatti et al., 2021). Cefiderocol total drug concentrations were determined only at the end of the dosing interval and corrected for protein binding of 58%. The authors arbitrarily defined exposures as sub-optimal (*f*Cmin/MIC <1), quasi-optimal (*f*Cmin/MIC 1- <4), or optimal (*f*Cmin/MIC ≥4). Microbiological failure occurred in 80% of patients with suboptimal fCmin/MIC compared with 29% of those achieving optimal or quasi-optimal fCmin/MIC ratio. The majority of failures occurred in patients with ventilator associated pneumonia.

Meropenem pharmacodynamics were evaluated in patients participating in clinical trials of lower respiratory tract infections (Li et al., 2007). The investigators used a covariate based population model to estimate meropenem pharmacokinetics and identified 54% *f*T > MIC to be associated with microbiological eradication. Notably, a *f*Cmin/MIC >5 was the only pharmacodynamic parameter significantly associated with clinical response, which was defined as cure or improvement of all signs and symptoms caused by the infection without additional antibiotic therapy. A population-based predictive model that evaluated the use of meropenem in adult patients with febrile neutropenia found an 80% clinical response rate when the *f*T > MIC was >75% (Ariano et al., 2005). Out of the 60 patients in the study, 42 clinical responders achieved an average exposure of 83% *f*T > MIC, whereas the average exposure for the 18 non-responders was 59% *f*T > MIC (*p* = 0.04). In 28 patients treated with meropenem, imipenem, or doripenem for *P. aeruginosa* ventilator-associated pneumonia, clinical response was 75% when the *f*T > MIC was 19.2%, and survival was 80% when >47.9% *f*T > MIC was achieved (Crandon et al., 2016). Kuti et al. (2018) analyzed the meropenem exposure in 15 children with cystic fibrosis (CF) acute pulmonary exacerbation to determine the pharmacodynamic threshold required for relative increased in forced expiratory volume (FEV_1_). Observed concentration profiles were observed for all participants and MICs were conducted by Etest. The majority of participants were infected *P. aeruginosa.* The meropenem *f*T > MIC was significantly associated $\mathrm{FE}V_{1}$ improvements post-treatment $\left[r^{2}=0.8,p<0.0004\right]$ (Kuti et al., 2018). Patients who achieved 65% *f*T > MIC had an increase in $\mathrm{FE}V_{1}$ ≥ 15% with a median increase in $\mathrm{FE}V_{1}$ of 28.5%. However, children who had <65% *f*T > MIC, had a median increase in $\mathrm{FE}V_{1}$ ≤ 7.8%.

A study that prospectively evaluated the *f*Cmin/MIC ratios of meropenem, piperacillin/tazobactam, ceftazidime, aztreonam, cefepime, and ceftriaxone in 98 patients with Gram-negative bloodstream infections identified a *f*Cmin/MIC >1.3 to be associated with clinical response (Wong et al., 2020). This exposure threshold was *f*Cmin/MIC >4.95 for meropenem, aztreonam, and ceftriaxone. While the investigators collected observed trough concentrations for all patients, MICs were conducted *via* a mix of Etest and VITEK AST, and the majority (71%) of patients had *f*Cmin/MIC ratios >5.

### Randomized Controlled Trials of TDM

To our knowledge, the results of three randomized controlled trials studying TDM of beta-lactam antibiotics are available at the time of writing. These studies may be useful to determine if the exposure thresholds sought by employing TDM achieved significant improvements in clinical outcomes. In one study, TDM was used to guide meropenem and piperacillin/tazobactam extended infusions in 41 critically ill patients (De Waele et al., 2014). The TDM target was 100% *f*T>4xMIC. Notably, very few patients achieved this aggressive exposure threshold with standard dosing. By 72 h, TDM patients were more likely to achieve the threshold (58% *versus* 16%, *p* = 0.007). However, no outcomes were reported in the study. A second clinical trial in 32 patients with neutropenic fever and hematologic malignancies aimed to achieve 100% *f*T > MIC *via* TDM for piperacillin/tazobactam (Sime et al., 2015). Like the first study, a greater percentage of patients in the TDM intervention arm achieved 100% *f*T > MIC (69% vs. 19%, *p* = 0.012). A greater number of patients also achieved 50% *f*T > MIC in the TDM arm (94 vs. 38%, *p* = 0.0001). However, no differences in clinical outcomes of fever or days to recovery from neutropenia were observed.

Finally, the TARGET study evaluated the role of TDM guided dosing for continuous infusion piperacillin/tazobactam in a multicenter, open-label randomized controlled trail of 249 adult patients with sepsis or septic shock (Hagel et al., 2021). Patients were randomized to TDM with goal exposure of 100% *f*T>4xMIC or *f*100% T > 64 if the pathogen or MIC were unavailable (i.e., 4 × 16 mg/L). Attainment of the pharmacodynamic threshold was more often achieved in patients with TDM; however, when adjusted for disease severity using baseline Sequential Organ Failure Assessment (SOFA) scores, the TDM-guided strategy for continuous infusion piperacillin/tazobactam did not result in greater resolution of organ dysfunction. Importantly, patients with steady state-concentrations >96 mg/L, as all well as those with concentrations <32 mg/L, had greater mortality compared with patients with concentrations absolute concentrations of 32–96 mg/L, which suggests there may be an upper limit to therapeutic range. Indeed, beta-lactams are known to have concentration dependent toxicities, particularly neurotoxicity, which is important to consider but outside the scope of this paper (Barreto et al., 2021).

### Exposure Response Relationships During Clinical Trials

The phase 3 randomized controlled registrational trials for beta-lactams are an ideal opportunity to confirm exposure response relationships and demonstrate adequacy of the dosage selected because clinical outcomes are rigorously defined, sparse pharmacokinetic sampling are routinely collected, and complete microbiology data are often available. Notably, some of these studies were unable to identify an exposure-response relationship. While this may be viewed as “unfortunate,” this is actually a positive observation, as it suggests the dosage developed from pre-clinical models was sufficient to achieve the murine based thresholds and result in successful clinical responses. Such studies are exemplified by ceftaroline in the treatment of community acquired pneumonia (Bhavnani et al., 2013) and cefiderocol (Kawaguchi et al., 2021) in the treatment of nosocomial pneumonia, blood stream infections, and complicated urinary tract infections. For these studies, the large majority of patients had *f*T > MIC exposures above the thresholds identified by pre-clinical murine studies and therefore no relationships with clinical study endpoints were identified. Additionally, during the cefiderocol analyses, no relationship with any of the study endpoints was observed even for 100% *f*T>4xMIC.

Ceftaroline exposure response relationships were identified during the phase 3 clinical studies of acute bacterial skin and skin structure infections (ABSSSI) (Bhavnani et al., 2015). Although failure rates were low in these studies, there was a robust spread of *f*T > MIC across the participants (range: 0–100%). As a result, classification and regression tree analyses identified 54.2 and 55% *f*T > MIC as thresholds predictive of microbiological success in all patients and those specifically with *S. aureus* infections, respectively. Microbiological success was 95.3% (486/510) for patients with *f*T > MIC of at least 54.2 compared with 68.8% (11/16) for those who did not achieve this threshold.

In contrast, ceftibiprole did not achieve pre-defined non-inferiority margins when studied for nosocomial pneumonia (Muller et al., 2014). Approximately 15% of patients (*n* = 52) contributed concentration data, while the remaining participants (*n* = 312) had their exposures estimated *via* a covariate based population model. Multiple logistic regression found *f*T > MIC to be associated with successful response. For patients with pathogens identified, 51% *f*T > MIC was identified as the threshold associated with eradication of the baseline pathogen by the end of treatment. Additionally, 62.2% *f*T > MIC was identified as threshold associated with eradication of any pathogen by end of therapy.

### Summary of Pharmacodynamic Thresholds From Clinical Studies

The majority of clinical studies (Table 1) suggest a pharmacodynamic threshold predictive of response that is congruent with pre-clinical murine studies (i.e., 40–70% *f*T > MIC). A few report higher threshold requirements (i.e., 100% *f*T>4xMIC or *f*Cmin/MIC >4); however, this may have been a result of too few patients achieving low *f*T > MIC in the trial or only evaluating Cmin/MIC exposures. Collectively, most clinical pharmacodynamic studies suffer from a number of limitations. Most notable include the use of covariate based population models to estimate the exposure instead of observed concentrations, small sample sizes, lack of full range MICs, mixed organisms and infection types, as well as inter-study variation in endpoint utilized (clinical success, microbiological eradication, mortality, etc), thus making systematic review/meta-analyses difficult. Future studies aimed at identifying pharmacodynamic thresholds for the beta-lactams should consider solutions to these limitations during study design.

**TABLE 1:** Summary of details for clinical pharmacodynamic studies of beta-lactams.

Country	Study design	# Of patients/status	Patient population	Bacteria	Drug(s)^a^	PK/PD target	Results	Strength	Weakness	References
United States	2 prospective RCT	76/sepsis and bacteremia	≥18 years	mixture, most common: *E.coli, P. aeruginosa, Klebsiella* spp.	FEP/CAZ	</≥ 80% *f*T > MIC; </≥ 100% *f*T > MIC	95.6%/97% had eradication of bacterial isolate in >80 and 100% *f*T > MIC	Clinical success seen in 73% of patients, with 89% having bacterial eradication	Power to detect a significance difference between clinical and microbiological outcomes was low for majority of infection types due to low sample sizes per subroup	McKinnon et al. (2008)
United States	Retrospective single-center	180/GNBSI	≥18 years with hematologic malignancy, neutropenia, kidney and renal transplant, mechanical ventilationetc.	Gram-negative isolates	FEP	>68%/>74% *f*T > MIC	92.9%/92.5% had higher rates of survival	Heterogeneous representation of organisms with FEP MICs between 1 and 64 mg/L; had patients with comorbid conditions	No plasma PK samples collected from outcome patients; bias due to study design; imputations of cefepime *f*T_>MIC_ were based on administrations within the first 24 h; no statistical differences between outcome groups according to infection source	Rhodes et al. (2015)
Canada	Retrospective single-center	248/FN	≥18 years with bacteremia	GPC and GNR	MEM	>75% *f*T > MIC	80% had clinical success rate	Study sample; majority reached target	Simulated plasma concentrations; predicted MIC values for isolates, study design; 60 patients included for PK/PD analysis	Ariano et al. (2005)
Australia	RCT single-center	32/FN	≥18 years with hematological malignancy	*B. cereus, E. coli, E. cloacae, K. pneumoniae, Staphylococcus* spp.	TZP II	100 *f*T > MIC/50% *f*T > MIC	69%/94% who had TDM reached targets	majority were able to hit target; PK/PD analysis	Did not evaluate clinical outcome or microbiological eradication; didn’t relate target with patient outcome	Sime et al. (2015)
Germany	Prospective single-center	48/ICU	≥18 years with sepsis, ARDS, and other conditions	GPC, *Pseudomonas* spp, *Bacillus* spp., *Clostridium* spp*.*, *Bacteroides* spp., *Mycoplasma* spp*.*, *Candida* spp. and *Aspergillus* spp	MEM	100% *f*T > MIC 2 mg/L + 8 mg/L/50% *f*T>4xMIC 2 mg/L + 8 mg/L	∼48.4%/20.6% for both targets at MICs of 2 mg/L and 8 mg/L, respectively	Developed graphical tool to predict risk of target non-attainment under MEM dosing based on ICU patient’s renal function	Did not use various MEM doses; small number of CRRT patients in study (7 patients)	Ehmann et al. (2017)
Netherlands	Prospective two-center	147/ICU	≥18 years with different conditions	*Enterobacterales, S. aureus, P. aeruginosa, E. coli*	6 β-lactams	100% *f*T > MIC/100% *f*T>4xMIC	63.3%/36.7 met target	large study; showed risk factors for patients who might not meet target (high BMI, eGFR ≥90 ml/min/1.73 $m^{2}$ , male gender)	MIC values from ECOFF values to calculate target attainment-value may not be accurate; higher number of males in study than females-target may not be based on gender	Abdulla et al. (2020)
Belgium	RCT single-center	41/SICU	≥18 years with arterial catheter	Enterobacteriaceae, *S. aureus, S. viridans*	TZP + MEM EI	100% *f*T > MIC/100% *f*T>4xMIC	95%/68% were able to achieve target with TDM	Used TDM and higher percentage of patients reached target	Impaired renal function or those on CRRT did not receive TDM-may be underdosed; Measured total and not free antibiotic concentrations	De Waele et al. (2014)
United States	Retrospective cohort study	56/ICU	≥18 years with non-urinary tract *P. aeruginosa* infections	*P. aeruginosa*	FEP	60% *f*T > MIC	77.8%/36.2% failed to achieve target when ≤60% *f*T > MIC and >60% *f*T > MIC	Measured microbiological response	study design-could not collect PK data for all patients; small sample size	Crandon et al. (2010)
Switzerland	Retrospective single-center	36/Burn Center	≥18 years	multiple, but majority *P. aeruginosa*	IMP + MEM	100% *f*T > MIC	38% did not reach target after first TDM	study sample; showed doses before and after TDM	TDM was performed in severely-ill patients; study design	Fournier et al. (2015)
United States	RCT	124/CABP	≥18 years	*S. pneumoniae, H. influenzae or H. parainfluenzae,* Enterobacteriaceae	CPT	100% *f*T > MIC	87.1%,91.1%/, and 98.4% had *f*T > MIC values of 100, 91.7, and 63.3%, respectively	High clinical and microbiological success rates (84.7 and 86.3%, respectively)	Failed to identify relationships between f%T > MIC values and responses	Bhavnani et al. (2013)
Japan	Retrospective analysis	516/pneumonia, BSI, cUTI	≥18 years	Gram-negative isolates	FDC	100% *f*T > MIC	>90% had 100% *f*T > MIC against MICs of ≤4 μg/ml for all infection sites and renal function groups, excepts for BSI/sepsis patients with normal renal function	Supports cefiderocol plasma exposure for patients with multiple illnesses	No relationship with any of the study endpoints was observed even for 100% fT > 4xMIC	Kawaguchi et al. (2021)
United States	Retrospective analysis	526/ABSSSI	≥18 years	MRSA	CPT	54.2 and 55% fT > MIC	94.7 and 94.5% had clinical and microbiological success, respectively/95.3 and 94.5% had clinical and microbiological success, respectively, when infected with *S. aureus*	Low failure rates	Robust spread of *f*T > MIC across the participants	Bhavnani et al. (2015)
United States	Retrospective cohort study	180/GNBSI	≥18 years old	Gram-negative isolates (mostly *E. coli*, *P. aeruginosa*, and *K. pneumoniae*)	FEP	68–75% *f*T > MIC	Cefepime exposures of 68–74% *f*T > MIC were associated with a higher odds of survival, survival improved if exposures were >64% and >74%	Sample size, survival endpoint, use of two covariate models to estimate cefepime PK independently	MICs derived from VITEK AST system with limited range between 1 and 64 mg/L	Rhodes et al. (2015)
Netherlands	Retrospective cohort study	154/nosocomial pneumonia	≥18 years old	Gram-negative bacteria	CAZ	>45% *f*T > MIC	Favorable outcome associated with >45% *f*T > MIC	Sample size; significant correlation between % *f*T > MIC during treatment and microbiological outcome as well as clinical outcome	Population PK model used to estimate exposures and not observed human concentrations	Muller et al. (2013)
United States	Retrospective PK/PD analysis	73/VAP	≥18 years of age	Multiple, but mostly *P. aeruginosa*	FEP,CAZ	53% *f*T > MIC	Microbiological success rate was 58.9%	Majority hit target when using antipseudomonal cephalosporins	Population PK model used to estimate exposures and not observed human concentrations	Macvane et al. (2014b)
Israel	Retrospective study	78/Bacteremia	≥18 years of age	*P. aeruginosa*	TZP	60% *f*T > MIC	>60.68% *f*T > MIC was associated with improved in-hospital survival and was statistically significant	>60.68% *f*T > MIC was associated with in-hospital survival	Covariate PK model used to estimate exposure and not observed human concentrations	Tannous et al. (2020)
United States	Prospective, open-label study	20/Gram-negative infections	≥18 years of age	Multiple Gram-negative bacteria	FEP	T > 4.3x MIC	Microbiological success was 89% when T > MIC was at least 100%, and 0% when it was less than 100% (*p* = 0.032); Using CART, Cmin/MIC ratio of 4.3 was significantly associated with microbiological success	Actual MICs of isolates were provided	range of exposures were not described	Tam et al. (2002)
United States	Retrospective study	33/pneumonia	≥18 years of age	Multiple Gram-negative bacteria, majority *p. aeruginosa*	FEP	100% *f*T > MIC	32/33 achieved this endpoint when an isolate had an MIC of 8 mg/L 12 patients had clinical failure when fCmin/MIC ratio <2.1	Majority of patients hit PK/PD target; provided MIC’s from VITEK	Cefepime concentrations were estimated based on patient-specific characteristics and not a direct measurement	Aitken et al. (2015)
Italy	Case series	13/ICU	≥18 years of age	*A. baumannii*	FDC	*f*C_min_/MIC ratio ≥4	Microbiological failure occurred in 80% of patients with suboptimal *f*Cmin/MIC (<1) compared with 29% of those achieving optimal (≥4)or quasi-optimal (1-<4) *f*Cmin/MIC ratio	The association between exposure and microbiological outcome was assessed	Power to detect a significance difference between clinical and microbiological outcome was low due to the low sample size	Gatti et al. (2021)
United States	Retrospective cohort study	101/LRTI	>17 years of age	Gram-negative and Gram-positive isolates, mostly *S. pneumoniae*	MEM	Not applicable	54% *f*T > MIC associated with microbiological eradication; *f*Cmin/MIC >5 associated with clinical response	88% of patients had clinical success	Actual patient concentrations are not provided	Li et al. (2007)
Canada	Retrospective, multi-center study	60/FN	≥18 years of age	Gram-positive and Gram-negative isolates, majority *S. epidermidis*	MEM	>75% T > MIC	70% of clinical responders achieved 83% *f*T > MIC; 30% of non-clinical responders achieved 59% *f*T > MIC	Majority of patients achieved the clinical outcome	Actual patient concentrations are not provided	Ariano et al. (2005)
United States	Retrospective analysis	86/VAP	≥18 years of age	*P. aeruginosa*	IMP, MEM, DOR	19.2%*f*T > MIC	75% had a clinical response when 19.2%*f* > MIC; Survival was 80% when >47.9% *f*T > MIC	Correlated PD with the development of carbapanem resistance in patients	Did not identify statistically significant PD associations for recurrence or resistance	Crandon et al. (2016)
United States	Retrospective, multi-center study	15/CF	<18 years of age	*P. aeruginosa*	MEM	>65% *f*T > MIC	Patients who achieved 65% *f*T > MIC had an increase in $\mathrm{FE}V_{1}$ ≥ 15%. However, children who had <65% *f*T > MIC, had a median increase in $\mathrm{FE}V_{1}$ ≤ 7.8%	Actual MIC data and observed patient concentrations were provided	All patients received a fluoroquinolone or an aminoglycoside; difficult to assess the effect of MEM exposure alone; concentrations and MIC data not collected for combination drugs	Kuti et al. (2018)
Belgium, Australia	Prospective study	98/GNBSI	≥18 years of age	Multiple Gram-negative isolates, mostly *E.coli*	FEP, CAZ, PZA, MEM, ATM, CTX	*f*Cmin/MIC >1.3	80% of patients achieved PK/PD target.	Collected observed trough concentrations for all patients	MICs were conducted via a mix of Etest and VITEK AST, and the majority (71%) of patients had *f*Cmin/MIC ratios >5	Wong et al. (2020)
Netherlands	Retrospective study	394/Nosocomial pneumonia	≥18 years	Gram-negative and Gram-positive isolates, including MRSA	BPR	51%*f*T > MIC	62.2% *f*T > MIC was associated with microbiological eradication; CART analysis showed 51.1% was associated with clinical cure	Isolates provided with MICs	Exposures were from a previous population PK model and individual PK data	Muller et al. (2014)

PK/PD, pharmacokinetic/pharmacodynamic; RCT, randomized controlled trial; LRTI, lower respiratory tract infection; GNBSI, Gram-negative bloodstream infection; FN, febrile neutropenia; ICU, intensive care unit; CF, cystic fibrosis; CRRT, continuous renal replacement therapy; SICU, surgical intensive care unit; KPC-KP, Klesbiella-pneumonia carbapanemase-producing Klesbiella *pneumoniae;* GPC, Gram positive cocci; GNR, Gram negative rods; ARDS, acute respiratory distress syndrome; MRSA, methicillin-resistant *S. aureus;* FEP, Cefepime; CAZ, ceftazidime; MEM, meropenem; TZP, piperacillin-tazobactam; II, intermittent infusion; CI, continuous infusion; EI, extended infusion; C_T, ceftolozane-Tazobactam; CPT, ceftaroline; IMP, imipenem; TDM, therapeutic drug monitoring.

a Not all cases were treated solely with β-lactams, but only the β-lactams used in the respective trial are included in this table.

It should also be noted that, by design, clinical pharmacodynamic studies are unable to parse pharmacodynamic parameters due to fixed dosages used during treatment. Because dose fractionation is not feasible, multiple pharmacodynamic indices often demonstrate statistical significance (Li et al., 2007; McKinnon et al., 2008; Muller et al., 2014; Kuti et al., 2018). It is our opinion that the pre-clinical models should set precedent on the index most predictive of effect, and the aforementioned clinical data are not sufficient to counter the robustness of dose ranging and dose fractionation study design utilized in pre-clinical models. Clinical studies are also not able to assess threshold variability by strain. Finally, the MIC itself is limited by routine two dilution testing and permitted variability of at least a dilution in each direction (i.e., 100% variability on each side of the MIC). This is further complicated in the clinical setting when the MIC is derived by automated susceptibility testing instead of a reference method and only once. These limitations on the MIC are in part why some have suggested much higher PD thresholds for TDM than observed during pre-clinical studies; however, once again, our review has not identified evidence of needing such high exposures for response. In contrast, such high exposure could lead to avoidable adverse events.

## Conclusion

Herein, we reviewed pre-clinical and clinical studies of beta-lactams with the aim of determining optimal exposure thresholds. These studies collectively support *f*T > MIC as the pharmacodynamic parameter associated with beta-lactam effect. Pre-clinical murine infection studies continue to report thresholds in the range of 40–70% *f*T > MIC for 1 log_10_ CFU reductions, with Gram-negatives typically requiring greater exposure compared with Gram-positives. In only a few murine studies were exposure thresholds greater than 70% *f*T > MIC. Furthermore, these thresholds have been successfully applied to optimize dosage development for use in clinical registrational studies, as well as for decision support in setting accurate susceptibility breakpoints.

While limitations in study design exist, the majority of clinical pharmacodynamic studies, including those conducted post-hoc on registrational trials, are supportive of pre-clinical thresholds. However, a small number of studies do report higher thresholds, and these studies are routinely referenced as justification for requiring excessive exposure (e.g., 100% *f*T>4x MIC). As a result, TDM guidelines and review articles continue to recommend high exposures for beta-lactams, while randomized controlled trials of TDM requiring such exposures have yet to demonstrate superiority.

In conclusion, our review of pre-clinical and clinical pharmacodynamic studies of the beta-lactams support exposure thresholds derived from drug specific pre-clinical murine studies to support optimal dosage selection and to guide susceptibility breakpoint assessments. Until further evaluated in antibiotic specific clinical studies, exposures of 50–100% *f*T > MIC are reasonable to define the beta-lactam therapeutic window for most infections.
